# Measurements of Volatile Organic Compounds in a Newly Built Daycare Center

**DOI:** 10.3390/ijerph13070736

**Published:** 2016-07-21

**Authors:** Miyuki Noguchi, Atsushi Mizukoshi, Yukio Yanagisawa, Akihiro Yamasaki

**Affiliations:** 1 Department of Materials and Life Science, Faculty of Science and Technology, Seikei University, 3-3-1 Kichijoji-kitamachi, Musashino, Tokyo 180-8633, Japan; noguchi@ejs.seikei.ac.jp; 2 Department of Environmental Medicine and Behavioral Science, Kindai University, 377-2 Ohno-higashi, Osakasayama 589-8511, Japan; mizukoshi@med.kindai.ac.jp; 3 Department of Environmental Systems, Institute of Frontier Sciences, The University of Tokyo, 5-1-5 Kashiwanoha, Kashiwa, Chiba 277-8563, Japan; yukio@kaiseigakuen.jp

**Keywords:** indoor air of new buildings, VOCs, TVOC, guideline value, target compounds, air exchange rate

## Abstract

We measured temporal changes in concentrations of total volatile organic compounds (TVOCs) and individual volatile organic compounds in a newly built daycare center. The temporal changes of the TVOC concentrations were monitored with a photo ionization detector (PID), and indoor air was sampled and analyzed by Gas Chromatography/Mass Spectrometry (GC/MS) and high performance liquid chromatography (HPLC) to determine the concentrations of the constituent VOCs. The measurements were performed just after completion of the building and again 3 months after completion. The TVOC concentration exceeded 1000 µg·m^−3^ for all the sampling locations just after completion of building, and decreased almost one tenth after 3 months, to below the guideline values of the TVOC in Japan at 400 µg·m^−3^. The concentrations of the target VOCs of which the indoor concentrations are regulated in Japan were below the guideline values for all the cases. The air-exchange rates were determined based on the temporal changes of the TVOC concentrations, and it was found that the countermeasure to increase the air exchange rate successfully decrease the TVOC concentration level in the rooms.

## 1. Introduction

Because of increasing concerns about the indoor air quality, reduction of volatile organic compounds (VOCs) that can harm human health in a variety of ways, including sick building syndrome (SBS), has been recognized as an urgent issue in many countries [1,2,3,4,5,6,7]. In Japan, the Ministry of Health, Labor and Welfare (MHLW) has selected eight target chemicals which guideline values in indoor air have been established, as shown in Table 1 [8]. These VOCs have been selected because of their known harmful health effects and the frequencies at which they have been detected in indoor air.

Various options for reducing indoor concentrations of VOCs have been attempted such as selecting building materials with low VOC emissions, and maintaining a suitable ventilation rate, especially for newly built buildings and houses. Recent surveys for new houses have demonstrated that indoor air concentrations of formaldehyde and toluene, which are major target compounds in the MHLW list, scarcely exceed the guideline values [9]. In spite of this, a considerable number of people have continued to complain about health problems probably due to indoor chemical substances in Japan [10,11,12]. These adverse health effects could be attributed to personal exposure to VOCs other than the target eight VOCs shown in Table 1.

To cover other VOCs that may be potentially harmful to human health, the guideline concentration of total VOCs (TVOC, hereafter) has been also established by MHLW in Japan at 400 μg·m^−3^ [8]. Although the guideline value for TVOC may not be necessarily be based on proven human health effects, there is no doubt that the reduction of the indoor TVOC concentrations would be desirable for preventing health effects such as SBS [6,13,14,15,16,17]. The TVOC concentration is defined as the total concentrations of components detected in the range between hexane (C_6_) and hexadecane (C_16_) on the chromatogram of an automatically thermal desorption gas chromatography/mass spectrometer (ATD-GC/MS) [6]. A number of studies have been published so far on the measurements of the VOC concentrations and the indoor air emission rate [11,12,18,19,20,21,22,23,24,25,26,27,28].

To reduce the indoor concentration of TVOC, there are two obvious measures: increasing the ventilation rate to dilute TVOC and reducing the emissions from the materials used. In Japan the Building Standards Act requires the installation of constantly running mechanical ventilation systems in newly built buildings to maintain an air exchange rate of at least 0.5 h^−1^ [29]. New buildings are occasionally designed to be more airtight than older houses, mainly for power saving purposes [30,31], and the airtight houses require higher air exchange rates to maintain the TVOC or VOC below the guideline level. Furnishings such as sliding doors and thick carpets often restrict the airflow, resulting in decreasing the effectiveness of the ventilation system [32,33]. Sometimes the ventilation systems are turned off by the occupants of the building.

The proper air exchange rate also depends on the emission rates of VOCs from materials used in the buildings. The air exchange rate at 0.5 h^−1^ would be sufficient to maintain the target VOC concentrations below the guideline level. In the reduction of TVOC levels by ventilation, however, we should consider emission sources of VOCs other than the target chemicals [34,35,36,37]. Emissions of VOCs would be naturally reduced with time due to the depletion of the sources, but it may take a period of time to reduce the indoor concentrations to acceptable levels. In the meantime, residents in newly built buildings may suffer adverse health effects such as sick building syndrome (SBS) [11,12,18,20,23,26,27,28,35]. For the reduction of concentrations of VOCs, we need to elucidate the emission rates of the constituent chemicals of TVOC, and the proper air exchange rate can be determined based on the emission rates of VOCs to maintain the indoor concentrations below the acceptable level.

Simultaneous measurements of the real-time monitoring and analysis of the sampled gas are crucial for the evaluation of the effectiveness of any countermeasures for TVOC reduction. Several kinds of equipment that enable the direct measurement and monitoring of the concentration of TVOCs have been developed. TVOC monitoring devices using a photo ionization detector (PID) are widely used equipment [38,39,40,41]. Continuous measurement of TVOC with PID would allow the monitoring of temporal change of TVOC concentrations, from which we can determine the air exchange or ventilation rates. The real-time monitoring of TVOC with PID could be useful for the evaluation of the effects of increasing ventilation on the TVOC reduction. Note that no regulations or guidelines have been established for TVOC concentrations using PID.

The purpose of this study was to characterize the indoor TVOC and individual VOCs in a newly built daycare center, and to determine the effective air exchange rate required to reduce indoor TVOC levels. For this purpose, we measured the temporal changes of TVOC concentration and the indoor concentrations of the constituent chemicals in a newly built daycare center in Kashiwa City, Chiba, Japan. Children’s exposures to indoor VOCs may be problematic [42,43,44,45]; SBS has been reported in some infants in a newly built schools or daycare centers, presumably due to high VOC concentrations [46,47]. SBS had been reported among children just after beginning of use of the daycare center, and countermeasures including installing an additional ventilation system was introduced to the daycare center which increased the ventilation rates. The measurements were performed just after completion and after 3 months with additional ventilation. We measured the TVOC concentrations with a PID-TVOC meter and the concentrations of the constituent chemicals in the sampled indoor air with chromatographic methods. The temporal change of TVOC concentration was used for evaluating the ventilation rate and the total emission rate of VOCs. We compared the concentrations of the constituent chemicals of the TVOC, and evaluated the emission from the materials used.

## 2. Experimental Section

### 2.1. Field Surveys

**Figure 1:**
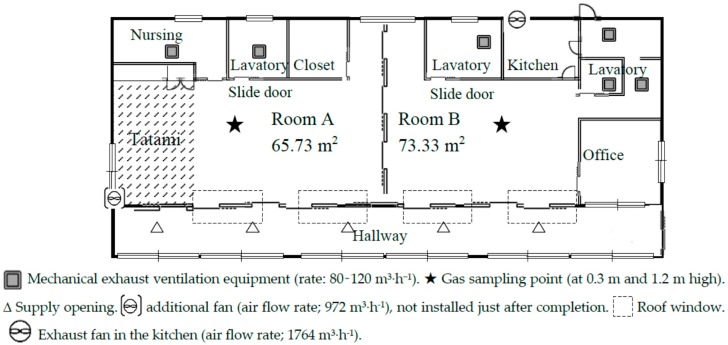
Floor plan of the new daycare center. The additional fan was not installed just after completion, and installed at the tatami room and operated at the measurements after 3 months.

Field surveys were performed in a newly built daycare center located in Kashiwa City, Chiba prefecture, located 30 km northwest of Tokyo, Japan. The building was handed to the occupants a few days after completion. The building contained constantly operating ventilation systems with exhaust fans as shown in Figure 1, where the floor plan of the daycare center is shown. There are two main rooms, namely room A (area: 65.73 m^2^) and room B (73.33 m^2^) in the daycare center. Room A has two mechanical exhaust ventilation fans and room B has four mechanical ventilation fans. The flow rates of these fans were in the range of 80 to 120 m^3^·h^−1^. The kitchen of room B was equipped with a strong exhaust fan, with an air flow rate of 1764 m^3^·h^−1^. Both rooms are connected to the hallway via roof windows. Indoor air is extracted mechanically through the outlets in the lavatories and the nursing room. Outdoor air is supplied naturally through the intake in the hallway. The lavatories and nursing room have sliding doors. The floor is wood flooring, except the tatami room, and the wall is made of plywood. The floor of the tatami room was covered by traditional Japanese straw matting. All the installed house furnishings were low-VOC-emission certified. The measurements of TVOC and air sampling were conducted at the gas sampling points shown by a star mark in Figure 1. In the room A, air was sampled at 1.2 m height. In room B, air was sampled at 2 heights (0.3 m and 1.2 m) from the floor. The height of 0.3 m corresponds to the breathing height of infants, and that of 1.2 m corresponds to that of adults. To prevent SBS among children in the daycare center, an additional fan with the air flow rate at 972 m^3^·h^−1^ was installed 3 months after (March of the next year) of the completion at the tatami area of room A to enhance the ventilation rate. The measurements were performed in December just after completion and after 3 month in March of the following year.

### 2.2. VOC and Carbonyl Compound Measurements

We measured the concentrations of VOCs and carbonyl compounds without occupants in the buildings from night time to the next morning. All of the windows of the building were opened to make the indoor concentrations of VOCs and carbonyl compounds equal to the outdoor concentrations. The windows were then closed, and the mechanical ventilation systems and fans were switched on. After 5 h, the active sampling of the indoor air was started. Thermal desorption tubes filled with Tenax TA (Supelco, Bellefonte, PA, USA) and Carboxen 1000 60/80 (Supelco) were used for collecting VOCs located at the sampling sites. The sampling time was 1 h, and the sampling rate was 100 mL·min^−1^. Thermal desorption-gas chromatography/mass spectrometry (ATD-GC/MS) was used for the analysis of the sampled components of VOCs in the sampling tubes. Carbonyl compounds were collected with 2,4-dinitrodiphenylhydrazine cartridges (XpoSure Aldehyde Sampler, Waters, Milford, MA, USA).

The flow rate was 1 L·min^−1^ and the sampling time was 1 h. The carbonyl compounds so collected were analyzed by high-performance liquid chromatography (HPLC) after extraction from the cartridges with acetonitrile. The equipment used and the analytical conditions are shown in Table 2. The TVOC concentration is determined by summing the concentration of the carbonyl compounds measured with HPLC and the total ion concentration detected in the GC/MS chromatogram, from C_6_ to C_16_, quantified as toluene. Simultaneously, measurements of TVOC concentrations were carried out with a photoionization detector (PID; ppbRAE with 10.6 eV UV lamp; RAE Systems, San Jose, CA, USA) [41]. The duration of the continuous measurement ranged from 10 h to 14 h depending on the case.

The TVOC value displayed by PID is based on isobutylene concentration. This value was converted to a toluene-based TVOC concentration by multiplying by the conversion factor (0.5) given by RAE [41]. Then the (toluene-based) mole fraction in ppb was converted to the mass concentration of TVOC in µg·m^-3^. It is known that the output of PID becomes unstable just after turning on the main switch. We waited for a certain time long enough for the PID to become stable before measurements. The effect of temperature change on the PID output was properly accounted for according to the instruction manual by using the measured temperature. The effect of relative humidity change was almost negligible because no noticeable change of relative humidity at the site was observed during the measurements.

The evaluation of the ventilation rates were carried out by the continuous measurements of the temporal change of the TVOC concentration by the PID TVOC meter at the room B of the daycare center just after completion and after 3 months. First, all the windows of the daycare center were opened to exchange whole indoor air with the outdoor air to confirm the TVOC concentration was zero. Then all the windows of the daycare center were closed, and the TVOC concentration was measured continuously to monitor the temporal change. In the meantime, the whole the ventilation system was operated and additional measures were conducted as usual. By this method, the ventilation rate without air exchange through the windows can be evaluated. This condition of the air exchange is consistent with the requirement by Japan Industrial Standard by using carbon dioxide [48].

Based on the temporal changes of the TVOC concentration measured with PID TVOC meter, the air exchange rate or the ventilation rate was estimated with the following method. Assuming the perfect mixing of the air in the room and the constant emission rate of TVOC, the concentration change of TVOC in the room can be expressed by the following Equation (1):

(1) $$C(t)=C_{i}+\left(\frac{E}{F}\right)(1-e^{-\frac{F}{V}t})=C_{i}+\left(\frac{E}{F}\right)(1-e^{-Nt})$$

where *C*(*t*) (μg·m^−3^) is the concentration of TVOC at time *t* (h), *C_i_*(μg·m^−3^) is the initial concentration of TVOC at *t* = 0, the time of closing windows, *E* (μg·h^−1^) is the emission rate of TVOC, *F* (m^3^·h^−1^) is the air exchange rate, and *V* [m^3^] is the room volume. *N* (h^−1^) is the air exchange per hour (ACH) given by, *N* = *F*/*V*. Equation (1) can be rearranged to:

(2) $$1-\{C(t)-C_{i}\}\left(\frac{F}{E}\right)=e^{-Nt}$$

Taking the logarithm of both sides of Equation (2) yields:

(3) $$\log\left[1-\{C(t)-C_{i}\}\left(\frac{F}{E}\right)\right]=-Nt$$

The initial concentration *C*_i_ can be determined from the experimental results. After a long time where the exponential term in Equation (2) can be assumed as 0, the concentration *C*(*t*) become constant. The steady state concentration, *C_st_* can be determined from the temporal change of the experimental results at which the concentration leveled off. Under the steady state condition, Equation (1) can be expressed by:

(4) $$C_{st}-C_{i}=\frac{E}{F}$$

Finally, Equation (3) can be expressed with one unknown parameter, *N*, the air exchange rate as:

(5) $$\log\left[\frac{C_{st}-C_{i}}{C_{st}-C(t)}\right]=Nt$$

Plotting left-hand side of Equation (5) against time, *t*, yields a straight line crossing the origin, and *N* was determined.

## 3. Results and Discussion

### 3.1. TVOC Concentration and VOCs Concentrations

**Figure 2:**
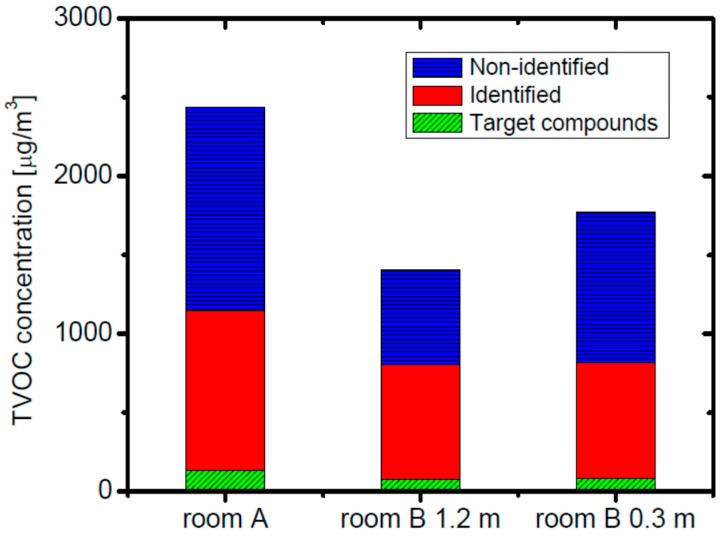
TVOC concentrations and compositions in rooms A and B (at 1.2 m and 0.3 m above the floor) just after the building was completed.

The results of the concentration measurements of the VOC and carbonyl compounds in indoor air sampled at the daycare center just after completion are shown in Table 3. The TVOC concentrations, measured with both methods, were much higher than the guideline value in Japan (400 μg·m^−3^) but the concentrations of all the target seven compounds detected were lower than the guideline values. The TVOC concentration at room A was higher than in room B, presumably because it had less mechanical exhaust ventilation equipment (two fans) than room B (which has four). Note that no additional fan was installed in room A during the measurements. The TVOC concentration at 0.3 m above the floor in room B was higher than that at 1.2 m above the floor in the room B. This is due either to the emission of VOCs from the floor or to a difference in the air flow patterns. The higher TVOC concentration at lower elevation may be especially problematic for babies and toddlers in the daycare center. The breakdown of the TVOC components based on the method using GC/MS and HPLC is shown in Figure 2. 

The target compounds indicate the sum of the concentrations of the target compounds in Table 1, and identified or non-identified indicates the total concentrations of the identified components and those of the unidentified components, respectively. The proportion of the target VOCs to the TVOC concentration was 6% at most for the case of room A, while identified compounds comprised 41% and unidentified compounds 53% of the TVOC concentration. Identified compounds that were found at high concentrations were ethyl acetate, d-limonene, α-pinene, and several kinds of carbonyl compounds. It can be considered that α-pinene was emitted from the wooden building materials, and ethyl acetate and butanone were emitted from the hydrolytic cleavage of the adhesive agents used in plywood.

Table 4 shows the results of the measurements of the TVOC, individual VOCs and carbonyl compounds three months after completion with additional ventilation measures. The TVOC concentrations three months later were approximately one tenth of the values measured just after completion. The TVOC concentrations as well as the concentrations of the seven target VOCs are below the guideline values for all the locations measured. Almost all of the VOC and carbonyl compound concentrations, except acetone, were reduced compared to the results just after completion of the building. The increase in the acetone level after three months could be attributed to some potential emission sources of acetone in the daycare center. However, we have not identified the emission sources at least during the measurement. In particular, 2-butanone, ethyl acetate, and d-limonene were much lower after 3 months. The measured concentrations of α-pinene were still high compared to other VOCs. This is due to the fact that wooden building materials were used to cover the entire surface of the daycare center rooms.

**Figure 3:**
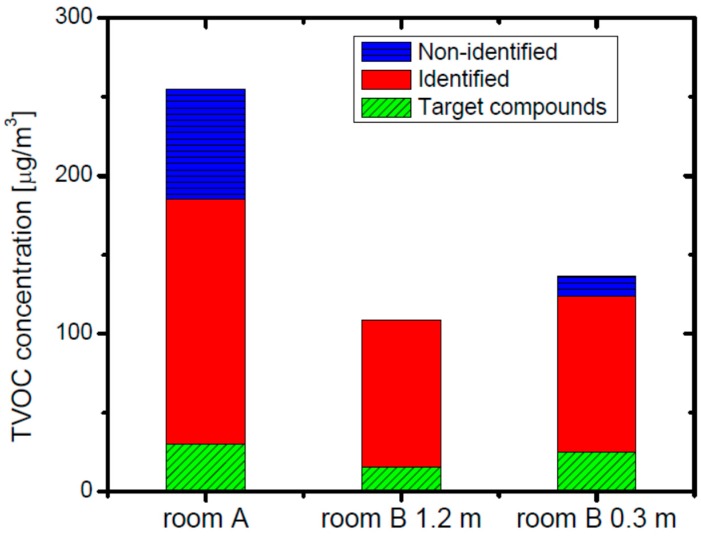
TVOC concentrations and compositions in rooms A and B (at 1.2 m and 0.3 m above the floor) 3 months after the building was completed.

The dramatic reduction of the concentrations of TVOC as well as those of the constituent VOCs could be attributed to the countermeasures implemented to increase the air exchange rate in the meantime in addition to the natural depletion of the emission sources. By the addition of an exhaust fan, the total ventilation rate was increased from 2364 m^3^·h^−1^ to 3336 m^3^·h^−1^ (about 40% increase at full operation). In addition the air exchange rate was intentionally increased by leaving the sliding doors of the lavatories and the nursing room approximately 15 cm open to allow air exchange from the connecting rooms in addition to the equipped mechanical exhaust system. The concentrations of VOCs at the lower height (0.3 m) at room B was generally higher than those at the higher height (1.2 m), which is similar to the results just after completion. Figure 3 shows the breakdown of the TVOC concentrations measured three months after completion. The proportion of unidentified components to the TVOC concentration was much lower than the cases just after the completion of the building shown in Figure 2.

### 3.2. Estimation of the Air Exchange Rate

**Figure 4:**
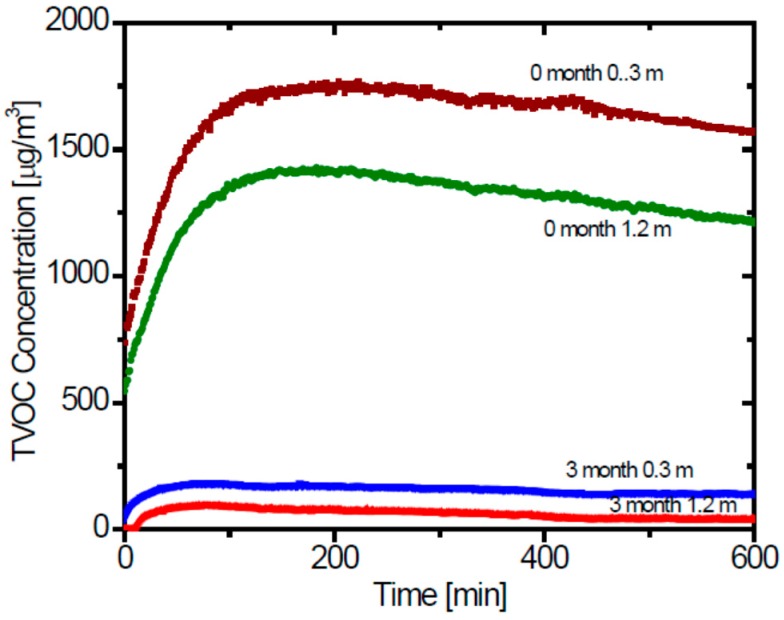
Time changes of the total volatile organic compound (TVOC) concentrations at Room B monitored using a PID after the windows had been closed, at just completion, and after three months.

Figure 4 shows the temporal changes of the TVOC concentration measured with the PID detector in room B, at two heights (0.3 m and 1.2 m). The concentration increased rapidly with time for the initial stage, and almost leveled off after a certain time for all the conditions.

**Figure 5:**
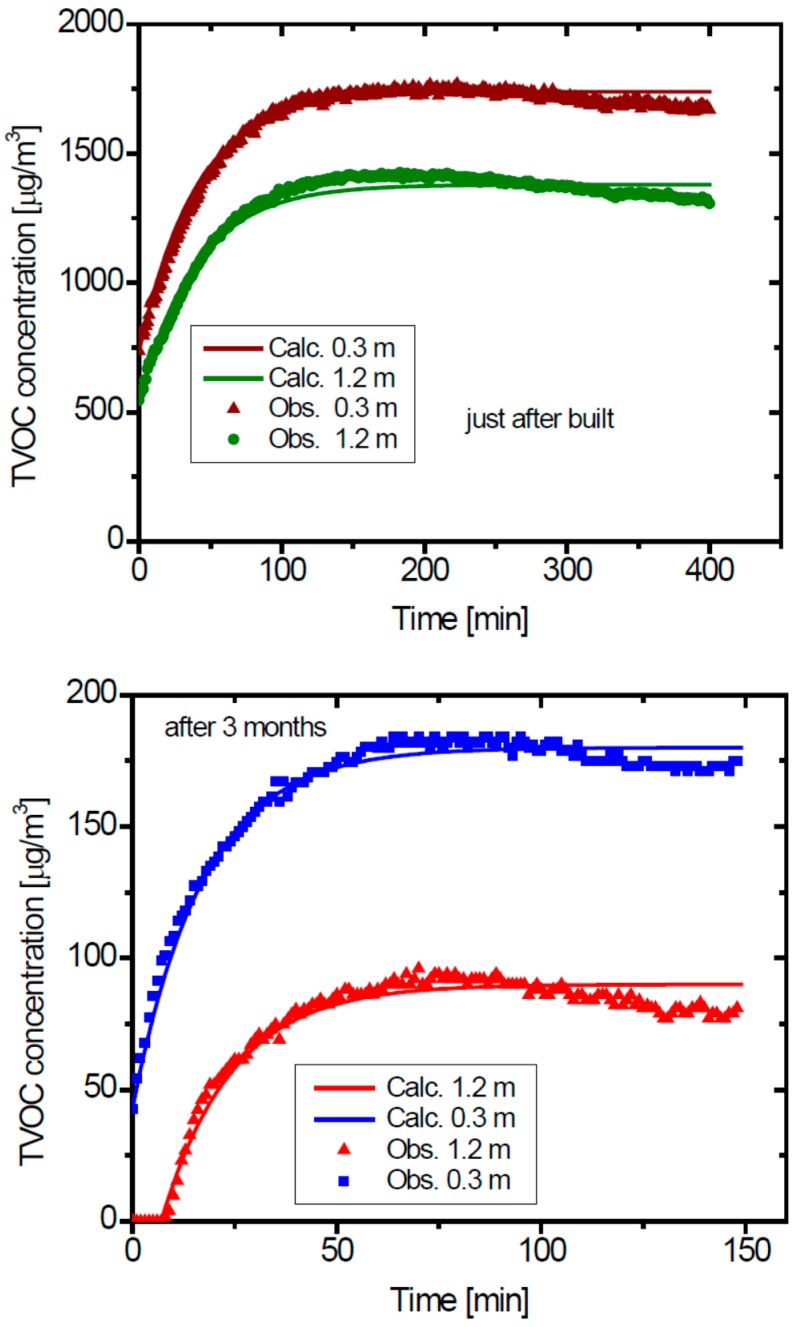
Curve fitted, using Equation (1), to the temporally changing TVOC concentrations measured just after completion.

Using the volume of room, *V* and determined value of *N*, we can obtain the air exchange rate, *F*, and the emission rate *E* from the results shown in Figure 4 based on the emission-ventilation Equations (1)–(5). First we determined the steady state concentration of each case from the figures. The results of the determined parameters are shown in Table 5. Figure 5 shows the results of the simulation of the temporal changes of the TVOC concentration. The simulation results of the temporal changes of the TVOC concentration agreed well with the experimental results for all the cases. The air exchange rates determined from the simulation are 1.5 h^−1^ for the case just after the completion of building, and 3.5 h^−1^ after 3 months applying increased air exchange measures. The increase in the air exchange rate was higher than the increase of the air flow rate by the newly added exhaust fan, which was expected to increase the air exchange rate at 40%, from 2364 m^3^·h^−1^ to 3336 m^3^·h^−1^. The increase in the air exchange rate can partially be attributed to intentional increase of the air exchange. The emission rates also decreased with time. Thus, the dramatic reduction of the TVOC concentration with time could be explained by both factors of the increased air exchanging rate and the reduction of the emission rate inside the house. These results would also demonstrate that the TVOC concentration in indoor air is a good indicator of indoor air quality and ventilation efficiency.

**Figure 6:**
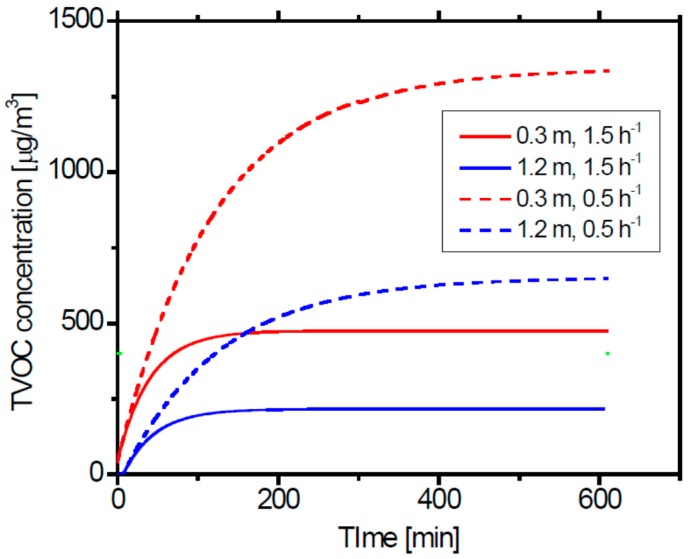
Simulation results of the TVOC concentration change with time for the cases of the air exchange rate was same as the one just after completion, but reduced air exchange rates of *N* = 1.5 h^−1^ and 0.5 h^−1^.

Figure 6 shows the simulation results of the temporal changes of the TVOC concentration for the case with the air exchange rate at 1.5 h^−1^ (the same as just after completion of the building) and 0.5 h^−1^ (regulation value of the Japanese Building Standards Act) with the emission rate being equal to the one after 3 months. The TVOC concentration exceeded 400 μg·m^−3^, the Japanese guideline value for TVOC, except in one case (high, 1.5 h^−1^). Thus, it is reasonable for this case to increase the air exchange rate to 3.5 h^−1^, otherwise the TVOC concentration would become higher than the acceptable level.

In this study, we measured the TVOC and individual VOC concentrations of only three samples twice with three month intervals. The results of the present study cannot be generalized to other cases. Our present results suggest that to determine when it is safe to occupy newly built buildings in terms of TVOC and individual VOC concentration levels, air samplings between 0 day and 3 months should be conducted to determine the time when the TVOC/VOC levels go below the guidelines.

## 4. Conclusions

The TVOC concentrations in a newly build daycare center were much higher than the guideline value of 400 μg·m^−3^ just after completion, although the concentrations of VOCs targeted in the Japanese Building Act were below the guideline values for all the components. Major contributors to the TVOC concentration were identified, but the major contributors were not the target compounds. The TVOC concentrations decreased with time and the TVOC concentration at three months were below half of the guideline value. The air exchange rates of the daycare center were estimated at 1.5 h^−1^ just after the completion of building, and were intentionally increased to 3.5 h^−1^ after three months. This increasing air exchange rate would be effective for the reduction of the TVOC concentration level in the daycare center. In conclusion we would suggest that the air exchange should be implemented with the maximum air exchange rate, during the initial 3 months after completion, and then decreased to an appropriate air exchange rate once the TVOC or individual VOC levels actually fall below the guidelines, in order to keep the levels acceptable.

## Figures and Tables

**Table 1:** Guideline indoor air values established by the Japanese Ministry of Health, Labour and Welfare for the target volatile organic compounds (VOCs).

Compound	Guideline Concentration (μg·m^−3^)
Acetaldehyde	48
*p*-Dichlorobenzene	240
Ethyl benzene	3800
Formaldehyde	100
Styrene	220
Tetradecane	330
Toluene	260
Xylenes	870

**Table 2:** Analytical conditions used for the automated thermal desorption-gas chromatography/mass spectrometry (ATD-GC/MS) and high-performance liquid chromatography (HPLC) systems.

ATD-GC/MS		
ATD		ATD 650 Turbo matrix (Perkin Elmer, Waltham, MA, USA)
	Primary desorption	300 °C (10 min)
	Secondary desorption	5 °C → 40 °C·min^−1^ → 300 °C (10 min)
GC/MS		HP6890/HP5973N (Agilent, Santa Clara, CA, USA)
	Column	HP-1 MS: 60.0 m length, 250-μm inner diameter, 1.00 μm film (Agilent, Santa Clara, CA, USA)
	Carrier gas	He, flow rate: 1 mL·min^−1^
	Column temperature	40 °C (4 min) → 7 °C·min^−1^ → 280 °C (10 min)
	Analytical mode	SCAN
	Mass range	*m*/*z* 33–550
**HPLC**	Instrument	HP1100 (Agilent)
	Column	Ascentis RP-Amide (250 mm length, 4.6 mm inner diameter, 5 μm particles (Sigma-Aldrich, St Louis, MO, USA)
	Mobile phase	H_2_O:CH_3_CN = 35:65
	Flow rate	1.0 mL·min^−1^
	Injection volume	20 μL
	Column temperature	35 °C
	Detector	Diode array detector 360 nm

**Table 3:** Volatile organic compound and carbonyl compound concentrations (μg·m^−3^) in air inside the daycare center just after completion.

	Concentration (μg·m^−3^)			
Compound	Room A, 1.2 m	Room B, 1.2 m	Room B, 0.3 m	Guideline Value
Formaldehyde *	9.5	7.4	7.0	100
Acetaldehyde *	33.0	23.0	21.1	48
Acetone	11.8	10.3	9.2	-
Propionaldehyde	8.8	5.9	6.0	-
Crotonaldehyde	37.7	129.0	128.1	-
Benzaldehyde	10.3	8.6	6.8	-
Isovaleraldehyde	22.3	13.3	11.4	-
*m*, *p*-Tolualdehyde	91.4	49.3	51.5	-
Hexaldehyde	104.8	63.5	57.0	-
2-Propanol	7.9	6.5	7.1	-
2-Butanone	161.6	155.4	159.0	-
Ethyl acetate	139.6	117.0	69.5	-
Benzene	10.3	8.2	6.2	-
Toluene *	16.9	11.2	10.7	260
*n*-Butyl acetate	12.2	15.6	23.2	-
Ethylbenzene *	6.4	8.3	10.7	3800
*m*, *p*-Xylene *	4.4	5.4	7.7	870
Styrene *	60.0	12.2	15.9	220
*o*-Xylene *	4.0	4.9	6.6	870
Nonane	5.6	2.5	3.1	-
α-Pinene	124.3	54.5	79.8	-
*p*-Ethyltoluene	20.9	7.9	10.7	-
*m*-Ethyltoluene	16.1	4.6	10.1	-
1,3,5-Trimethylbenzene	6.4	7.5	8.9	-
Decane	54.3	16.6	25.9	-
d-Limonene	154.1	49.1	50.6	-
TVOCs (GC/MS)	2327	1218	1589	400
TVOCs (PID)	2600	1206	1668	

* Target compound listed in Table 1.

**Table 4:** Volatile organic compound and carbonyl compound concentrations (μg·m^−3^) in air inside the daycare center after three months of completion.

	Concentration (μg·m^−3^)			
Compound	Room A, 1.2 m	Room B, 1.2 m	Room B, 0.3 m	Guideline Value
Formaldehyde	6.30	6.01	7.06	100
Acetaldehyde	9.48	8.75	9.74	48
Acetone	15.8	15.2	12.1	-
Propionaldehyde	<5.0	<5.0	<5.0	-
Crotonaldehyde	<5.0	<5.0	<5.0	-
Benzaldehyde	<5.0	<5.0	<5.0	-
Isovaleraldehyde	<5.0	<5.0	<5.0	-
*m*, *p*-Tolualdehyde	<5.0	<5.0	<5.0	-
Hexaldehyde	10.0	9.58	11.3	-
2-Propanol	3.66	3.15	1.28	-
2-Butanone	-	-	-	-
Ethyl acetate	-	-	-	-
Benzene	1.29	<0.2	<0.2	-
Toluene	9.63	<0.2	7.54	260
*n*-Butyl acetate	1.18	<0.2	<0.2	-
Ethylbenzene	0.687	<0.2	<0.2	3800
*m*, *p*-Xylene	1.11	<0.2	<0.2	870
Styrene	2.39	<0.2	<0.2	220
*o*-Xylene	0.687	<0.2	<0.2	870
Nonane	1.44	0.25	0.35	-
α-Pinene	82.4	34.1	40.1	-
*p*-Ethyltoluene	1.12	<0.2	0.22	-
*m*-Ethyltoluene	1.09	3.87	4.56	-
1,3,5-Trimethylbenzene	0.934	0.311	0.470	-
Decane	-	-	-	-
d-Limonene	11.3	0.876	3.17	-
TVOCs (with GC/MS)	201	48.5	82.0	400
TVOCs (with PID)	110	170	200	

**Table 5:** Air exchange rates and total volatile organic compound (TVOC) emission rates calculated using Equation (1).

Time of Measurement	Position Measured	Air Change per Hour (ACH), *N* = *F*/*V* (h^−1^)	Emission Rate, *E* (mg·h^−1^)
Just after completion	0.3 m high	1.5	700
	1.2 m high	1.5	584
After three months of completion	0.3 m high	3.5	63
	1.2 m high	3.5	126
